# Effects of marijuana and tobacco on male fertility and their relationship to genetic variation of mitochondrial cytochrome C oxidase genes

**DOI:** 10.1038/s41598-025-91894-0

**Published:** 2025-03-04

**Authors:** Houda Amor, Ayham Ismaeil, Peter Michael Jankowski, Mohammad A Al Smadi, Mazhar S Al Zoubi, Ingolf Juhasz-Böss, Mohamad Eid Hammadeh

**Affiliations:** 1 Department of Obstetrics, Gynecology & Reproductive Medicine, Biochemistry & Molecular Biology of Reproductive Medicine Laboratory, Medical Faculty, University of Saarland, Saarbrücken, Germany; 2 Department of Obstetrics & Gynecology, Department Endocrinology & Reproductive Medicine, University clinics Freiburg, Freiburg im Breisgau, Germany; 3 Prince Rashid Bin Al Hassan Hospital, Military Royal Force Hospital (PRBH) Irbid, Irbid, Jordan; 4 Department of Basic Medical Sciences, Faculty of Medicine, Yarmouk University, Irbid, Jordan

**Keywords:** Male infertility, Tobacco smoking, Cannabis, MtDNA, Cytochrome C oxidase genes, Reproductive disorders, Infertility

## Abstract

Although tobacco smoking declined among men at reproductive age, the use of cannabis increased. The aim of our study was to determine the impact of tobacco and cannabis on sperm quality, sperm DNA integrity tested by Chromomycin A3 (CMA3) and acridine orange (AO) and their association to genetic variants in the Cytochrome C Oxidase 1, 2 and 3 genes (*MT-CO1*, *MT-CO2*, and *MT-CO3*). Semen samples were collected and divided into 37 non-smokers (NS), 39 tobacco smokers (TS), and 37 cannabis smokers (CS). *MT-CO1*, *MT-CO2 and MT-CO3* genes were amplified by PCR and sequenced by Sanger. The results showed reductions in normal sperm morphology and non-progressive motility in TS and CS compared to NS (*p* < 0.001). However, immotile sperm, AO+, and CMA3 + scores were higher in the CS compared to TS and NS (*p* < 0.001). Twenty-three nucleotide substitutions (SNPs) detected in the *MT-CO1* gene, 15 SNPs detected in the *MT-CO2* gene, and 30 SNPs detected in the *MT-CO3* gene. None of these SNPs was different between the three groups. Tobacco and cannabis smoking altered the motility and morphology of the spermatozoa and sperm DNA integrity but was not associated with genetic variants in the *MT-CO1*, *MT-CO2* and *MT-CO3* genes.

## Introduction

At least about 14% of population in reproductive age suffers from infertility^1^. In fact, different pathological factors could affect male fertility. Commonly, 50% of subfertility cases are due to idiopathic infertility. Male infertility may be caused by genetic factors, such as abnormal karyotype^2^ and genetic defects such as single nucleotide polymorphisms (SNPs)^3^. Moreover, environmental and lifestyle factors especially diet, obesity, smoking, alcohol intakes, and recreational drugs use showed to have negative effect on male fertility^1^.

This has drawn the attention to the impact of tobacco and cannabis smoking on male reproductive health due to increased use of these substances worldwide. Understanding their potential impact on fertility is also crucial for the well-being of future generations.

Tobacco smoke is comprised of numerous toxic and mutagenic compounds and their resultant effect on organs like lungs and urinary bladder and their effects on male fertility has been documented. Notably, nicotine and its primary metabolite cotinine can traverse the blood-testis barrier, subsequently inflicting varying degrees of damage upon germ cells^4^. Tobacco smoking was suggested to be related to reductions in sperm concentration, abnormal sperm morphology, and altered protein expression in addition to genetic and epigenetic anomalies within spermatozoa^5^. Furthermore, some studies have substantiated the notion that tobacco smoke can act as both a mutagen and an aneugen within germ cells/spermatozoa ^6,7^.

In addition, cannabis is one of the most widely used recreational drugs and has been subject to significant reclassification and medical application. In 2004, it was downgraded from a Class B to a Class C drug. Its potential use in treating multiple sclerosis, alongside synthetic THC (Dronabinol) which is used in the United States and several European countries to alleviate AIDS-related cachexia, reduce nausea from cancer chemotherapy, and address chronic pain and anxiety, has fueled widespread misconceptions about its legality and health impacts^8^. Marijuana is the most widely used recreational drug, containing THC, which can negatively impact normal reproductive functions^9^. As marijuana legislation is passed in several countries and states, public health concerns are growing as it affects not only users but also their offspring. Researchers from Duke University (North Carolina) have demonstrated that cannabis consumption can modify the DNA methylation profile of exposed sperm^10^. A study conducted on male individuals who consumed cannabis within 90 days before providing a semen sample, aged ≤ 30 years, exhibited a higher likelihood of abnormal sperm morphology, motility, functionality, and quality^11–15^. The Cannabinoid receptor 1 (CB1) has demonstrated connections to mitochondrial activity within sperm, which is adversely influenced by cannabis exposure, culminating in compromised sperm locomotion. Although in vitro examinations have revealed plausible mechanisms, it remains uncertain whether these consequences are entirely mirrored in the male testicular environment^12^.

Sperm motility represents a critical factor for spermatozoa progression towards the oocyte and subsequent successful fertilization. The mitochondria, commonly acknowledged as the cellular powerhouse, possess their distinct genome encoding 13 proteins^16^. The mitochondrial genome plays a pivotal role in mature sperm morphogenesis and flagellar motility following ejaculation^17,18^. Mutation rates within mitochondrial DNA (MT-DNA) are relatively high, attributed to the absence of histones and DNA repair mechanisms^19^. Consequently, mutations that arise within the mitochondrial genome contribute significantly to particular genetic disorders. It has been documented that MT-DNA mutations correlate with specific forms of male infertility, such as POLG locus mutations in MT-DNA polymerase^20^. Additionally, a high prevalence of single-nucleotide polymorphisms (SNPs) within MT-DNA has been observed in semen samples resulting in poor sperm quality^21–24^.

*MT-CO1*, *MT-CO2*, and *MT-CO3* are core subunits of the cytochrome c oxidase (complex IV) localized in the inner mitochondrial membranes. More than 30 genes are correlated with the deficiency of COX abnormalities such as Leber’s Hereditary Optic Neuropathy LHON, hypotonia, optic atrophy, myopathy and lactic acidosis. With the other accessory subunits, the *MT-CO1*, *MT-CO2*, and *MT-CO3* represent the catalytic function of the COX complex^25^. A recent systematic review demonstrated the role of mtDNA genetic alteration in the development of male infertility^26^. However, most of the reported genetic alterations do not investigate the three core subunits in the COX complex. Limited studies investigated the genetic alterations of *MT-CO1*, *MT-CO2*, and *MT-CO3* in infertile men. For instance, a study reported an association between *MT-CO3* 15 bp deletion and male infertility^27^.

This study aims to determine the effects of tobacco and cannabis smoking in association with particular paternal mitochondrial genetic variants on spermatozoa function. Namely, we focused on studying the sperm mitochondrial genetic variants in the Cytochrome C Oxidase 1, 2 and 3 genes (*MT-CO1*, *MT-CO2*, and *MT-CO3*), and investigated the possible relationship with standard sperm parameters, spermatozoa DNA integrity and protamination.

## Results

### Smoking (Tobacco and Cannabis) and its correlation to sperm parameters and DNA quality

The study population included three groups: non-smoker individuals (NS, *N* = 37), tobacco smoker individuals (TS, *N* = 39), and cannabis smoker individuals (CS, *N* = 37).

**Fig. 1:**
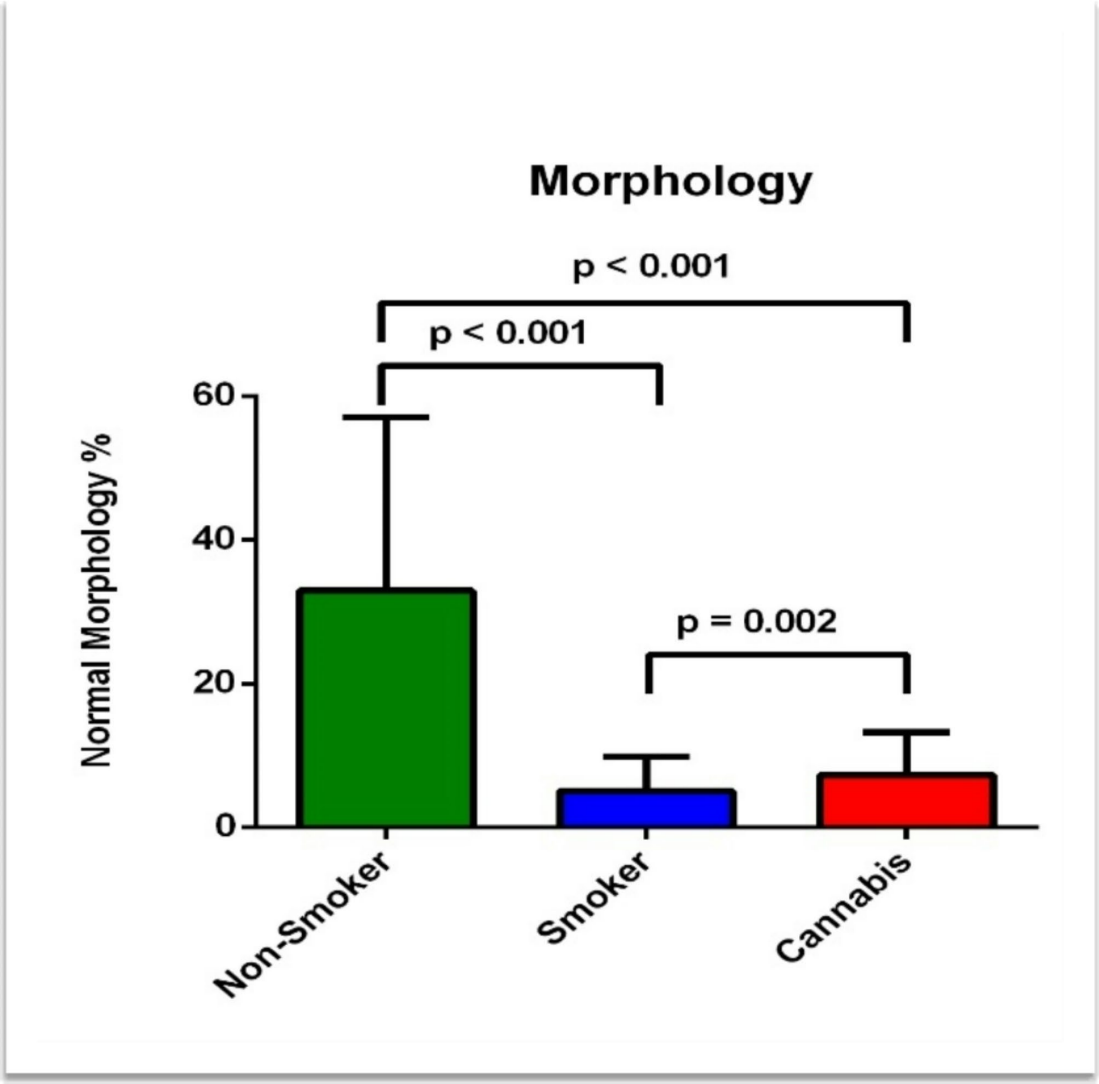
Difference of the mean morphologically normal sperm percentage between the three groups.

The semen analysis included sperm morphology, volume, motility, and concentration. In addition, AO, and CMA3 tests were performed in the three study groups. The results showed a significant reduction in normal sperm morphology in tobacco smokers (5.02 ± 4.8%) and cannabis smokers (2.26 ± 2.3%) groups compared to the non-smoker group (7.46 ± 5.9) (*p* < 0.001) (Table 1). Moreover, there was a significant reduction in normal sperm morphology in the cannabis smoker group compared to the tobacco smoker group (*p* = 0.002) as shown in Fig. 1.

**Table 1:** Comparison of the semen parameters between non-smokers, tobacco-smokers and cannabis smoker groups.

Parameters	NS (Mean ± SD)	TS (Mean ± SD)	CS (Mean ± SD)	One Way-ANOVA *P*-Value
Age (Years)	35.13 (± 7.7)	32.95 (± 6.3)	28.05 (± 3.7)	0.061
Volume (ml)	3.37 (± 1.2)	3.47 (± 1.4)	3.05 (± 0.8)	0.091
Sperm concentration (10^6^/ml)	33.86 (± 24.1)	30.65 (± 21.6)	28.37 (± 18.2)	0.199
Morphology (%)	7.46 (± 5.9)	5.02 (± 4.8)	2.26 (± 2.3)	< **0.001**
PR Motility (%)	14.27 (± 11.3)	13.12 (± 10.6)	10.18 (± 10.6)	0.223
NP motility (%)	34.40 (± 14.3)	27.82 (± 16.6)	20.63 (± 12.6)	< **0.001**
Immotile (%)	14.27 (± 11.3)	13.12 (± 10.6)	10.18 (± 10.6)	< **0.001**

**Fig. 2:**
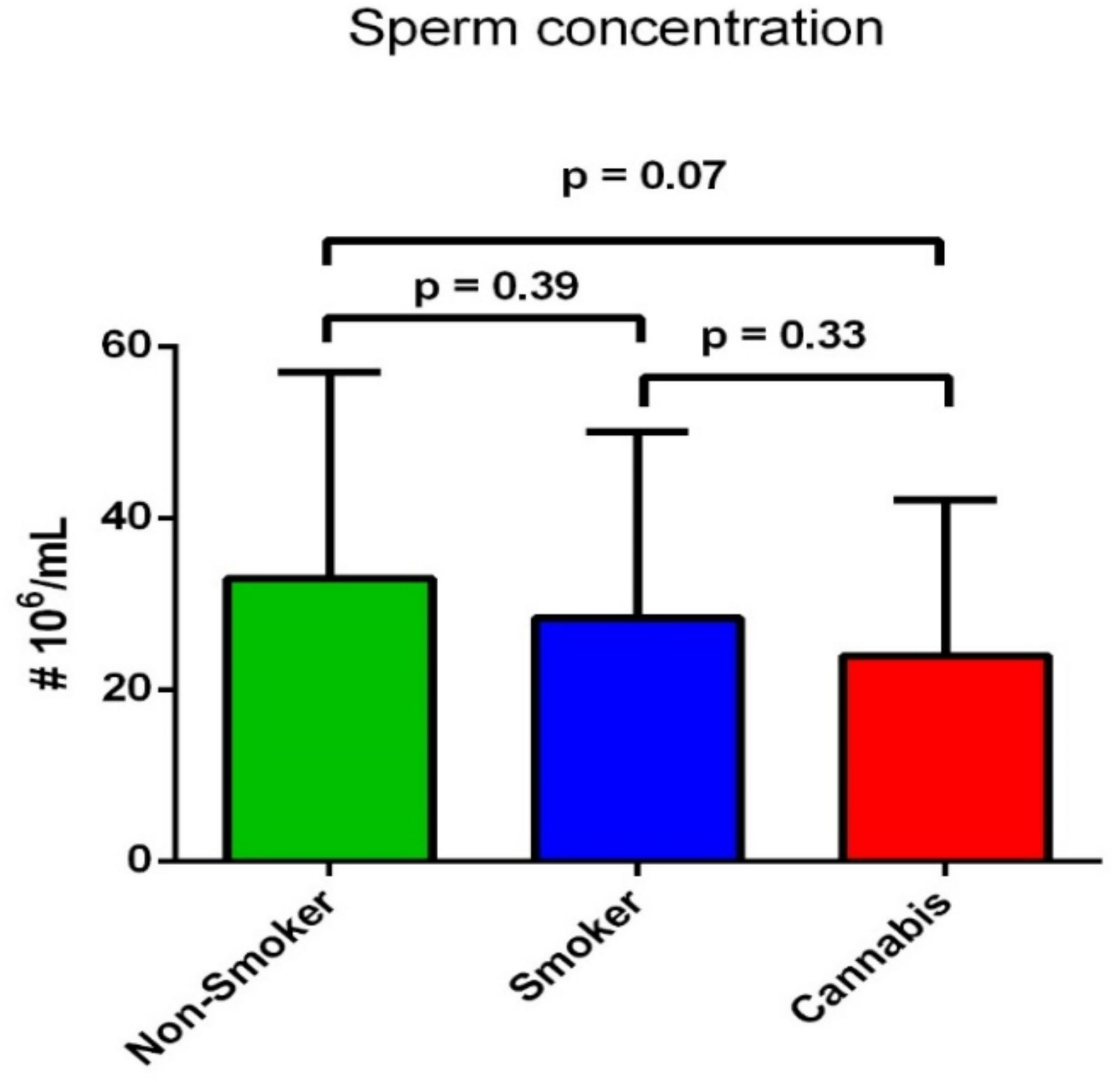
Difference in the sperm concentration (x10^6^/ml) between the three groups.

In addition, sperm concentration showed no significant difference between the three groups (*p* = 0.199). It showed a non-significant tendency of reduction in the cannabis-smoking groups (28.37 ± 18.2 × 10^6^/ml) compared to the non-smoking group (33.86 ± 24.1 × 10^6^/ml) (*p* = 0.07). At the same time, there was no significant difference in sperm concentration between the tobacco-smoking (30.65 ± 21.6 × 10^6^/ml) and cannabis-smoking groups (*p* = 0.33) or tobacco-smoking and non-smoking group (*p* = 0.39) as shown in Fig. 2. In addition, semen volume did not show significant differences between groups (*p* = 0.091) as shown in Table 1.

**Fig. 3:**
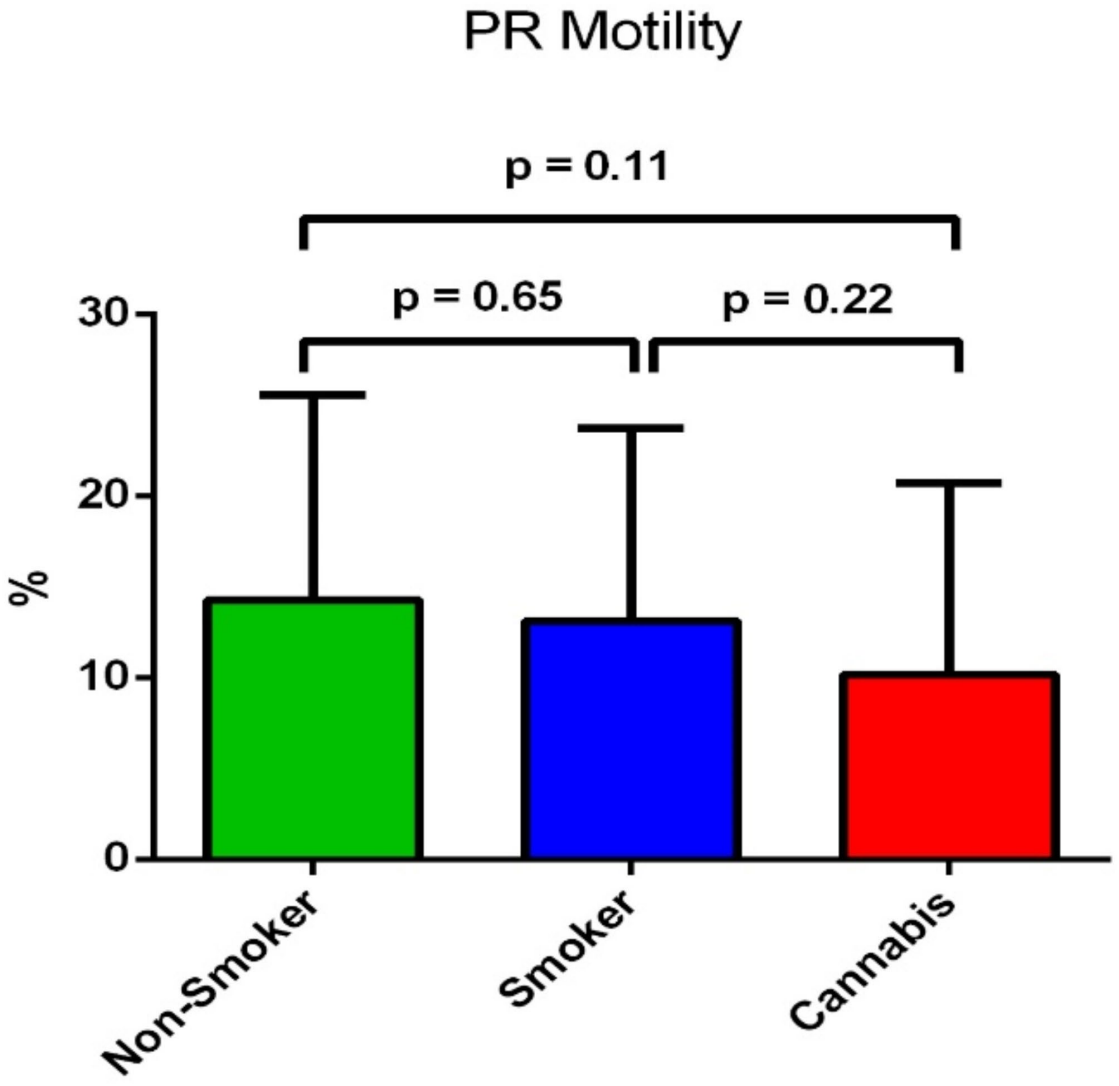
Difference of the sperm mean progressive motility percentage between the three groups.

Sperm total sperm motility was significantly different between the three groups (*p* = 0.008.). However, sperm progressive motility showed a non-significant difference between the three groups (*p* = 0.223) (Table 1). It showed a non-significant tendency of reduction in the cannabis-smoking groups (10.18 ± 10.6%) compared to the tobacco-smoking group (13.12 ± 10.6%) (*p* = 0.22), and to non-smoking group (14.27 ± 11.3%) (*p* = 0.11) as shown in Fig. 3.

**Fig. 4:**
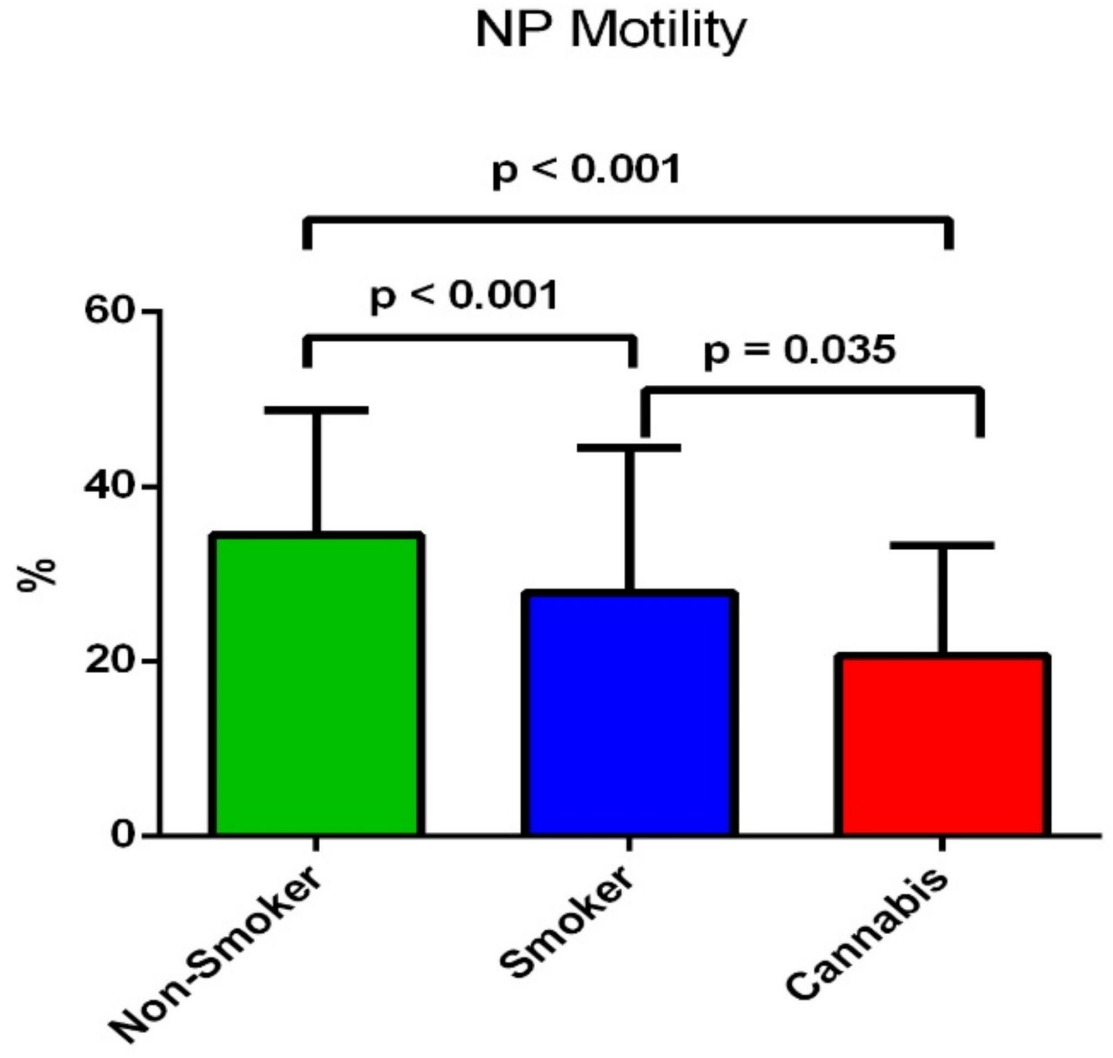
Difference of the sperm mean non-progressive motility percentage between the three groups.

Moreover, non-progressive motility showed a highly significant difference between the studied groups (*p* < 0.001) (Table 1). It demonstrated a significant reduction in the cannabis-smoking group (20.63 ± 12.6%) compared to the tobacco-smoking group (27.82 ± 16.6%) (*p* = 0.035) and to the non-smoking group (34.40 ± 14.3%) (*p* < 0.001) (Fig. 4).

In contradiction, the mean percentage of immotile sperm was significantly higher in the cannabis-smoking group (68.66 ± 21.9%) compared to tobacco smoking (58.92 ± 24.4) and the non-smoking group (51.73 ± 18.8%) (*p* < 0.001) (Table 1).

**Table 2:** Comparison of sperm DNA fragmentation assessed by AO staining (AO+) and the grade of Protamine deficiency in sperm DNA assessed by Chromomycin-A3 staining (CMA3+) between NS, TS, and CS.

Parameters	NS (Mean ± SD)	TS (Mean ± SD)	CS (Mean ± SD)	One Way-ANOVA *P*-Value
AO+ (%)	10.1 (± 14.2)	6.4 (± 10.2)	28.53 (± 15.8)	< **0.001**
CMA3+ (%)	15.0 (± 15.4)	25.3 (± 14.9)	37.13 (± 20.1)	< **0.001**

**Fig. 5:**
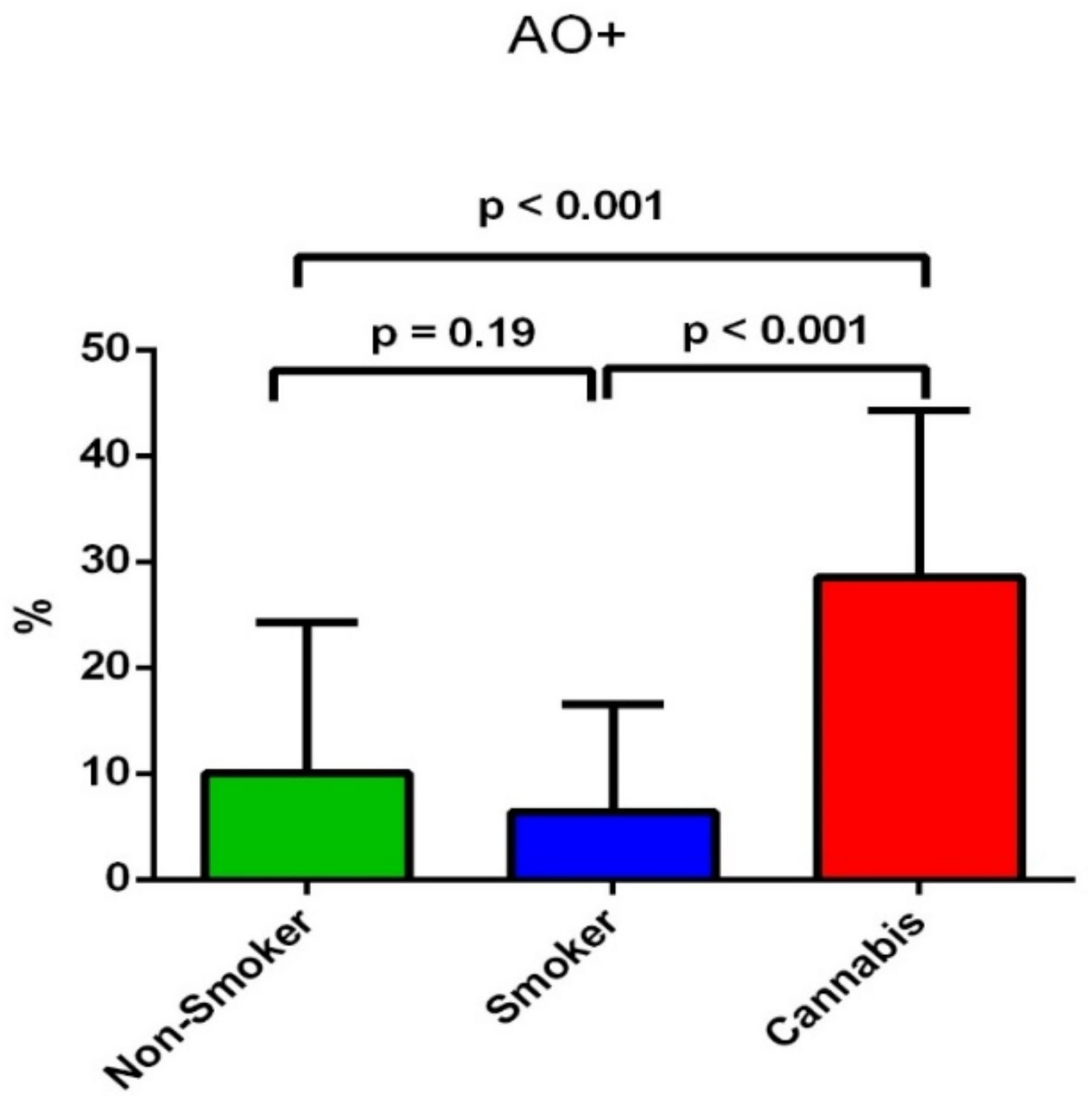
The difference of the the proportion of spermatozoa with DNA denaturation assessed by acridine orange test between the three groups.

DNA integrity was measured using an Acridine Orange (AO) assay and Chromomycin Staining (CMA3). The results showed a significant increase in the AO + score in the cannabis-smoking group (28.53 ± 15.8%) compared to the non-smoking group (10.1 ± 14.2%) and the tobacco-smoking group (6.4 ± 10.2%) (*p* < 0.001) (Table 2). At the same time, there was no significant difference in the AO + score between the tobacco-smoking and non-smoking groups (*p* = 0.19) as shown in Fig. 5.

**Fig. 6:**
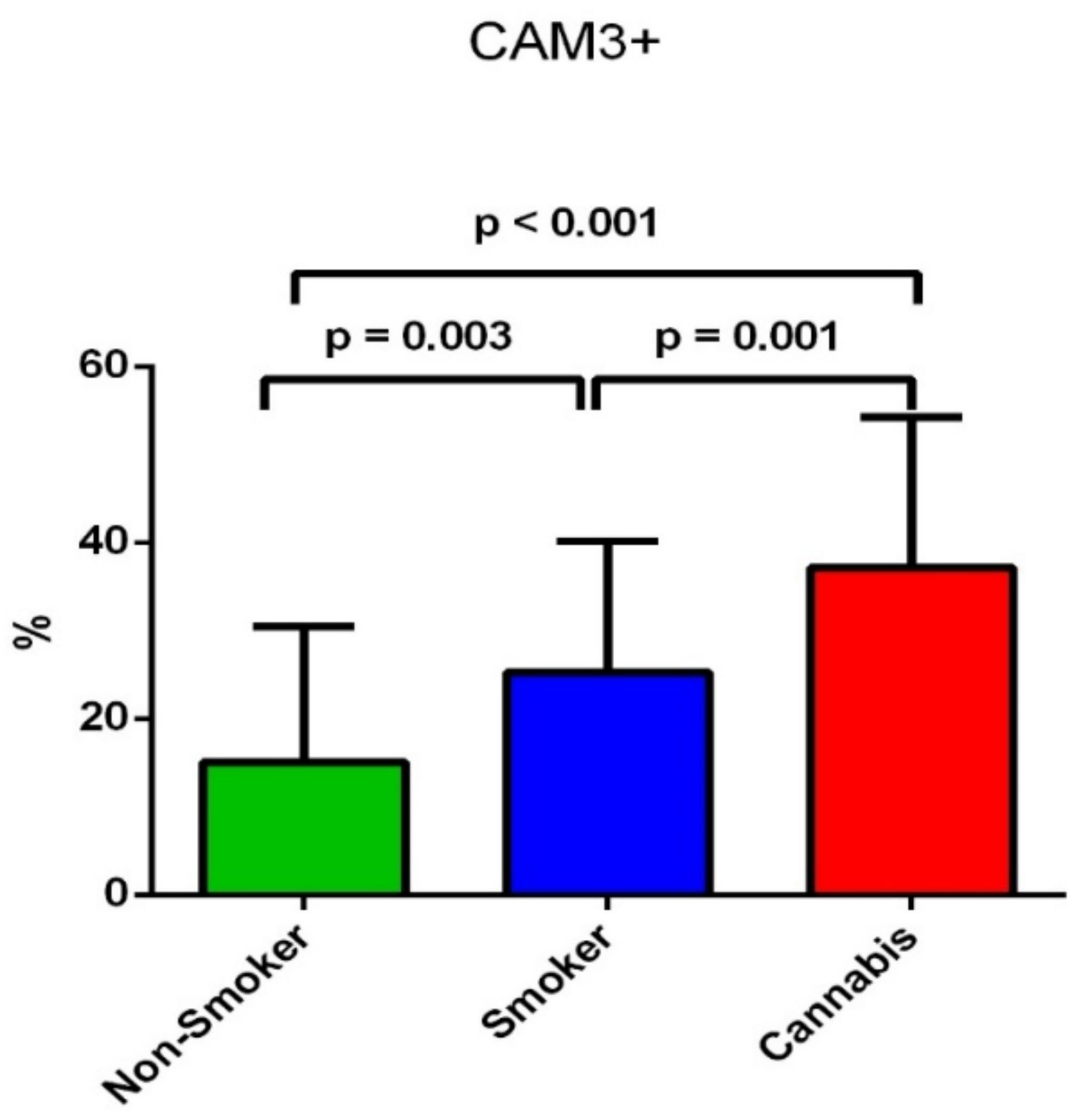
The difference of the proportion of spermatozoa with defective chromatin protamination assessed by Chromomycine CMA3 test between the three groups.

In addition, the CMA3 + test showed a significant increase in the CMA3 + score in the cannabis-smoking group compared to the non-smoking group (*p* < 0.001) and between the tobacco-smoking group compared to the non-smoking group (*p* = 0.003) (Table 2). At the same time, the CMA3 + score was significantly higher in cannabis-smoking men in comparison to tobacco-smoking men (*p* = 0.001) as shown in Fig. 6.

### *MT-CO1*, *MT-CO2* and *MT-CO3* SNPs distribution between non-smokers, tobacco smokers and cannabis smokers

The percentages of investigated men with total variants in the *MT-CO1* gene groups NS, TS, and CS were: 97.2%, 82.0%, and 86.4%, respectively (Table 3). However, none of these SNPs were significantly different between these groups (*p* = 0.10). A total of 23 single nucleotide substitutions (SNPs) in the mitochondrial cytochrome c oxidase subunit 1 (*MT-CO1*) were identified; 15 of them were synonymous variants, while eight were missense mutations (Table 4).

**Table 3:** The percentage of men with total mitochondrial variants in *MT-CO1*, *MT-CO2*, and *MT-CO3* among non-smokers, tobacco-smokers and cannabis smoker’s groups.

Gene	Non-Smokers (*N* = 37) Number of males with total variants (%)	Tobacco-Smokers (*N* = 39) Number of males with total variants (%)	Cannabis-Smokers (*N* = 37) Number of males with total variants (%)	Chi-square	*P*-Value
*MT-CO1*	36 (97.2%)	32 (82.0%)	32 (86.4%)	4.5	0.10
*MT-CO2*	7 (18.9%)	13 (33.3%)	7 (18.9%)	2.9	0.23
*MT-CO3*	25 (67.6%)	17 (43.6%)	17 (45.9%)	5.2	0.07

A total of 15 genetic alterations in the mitochondrial cytochrome c oxidase subunit 2 (*MT-CO2*) were identified; ten of them were synonymous variants and five of them were missense mutations (Table 5). The percentage of men with total variants in the *MT-CO2* gene groups NS, TS, and CS were: 18.9%, 33.3%, and 18.9% respectively (Table 3). None of these SNPs were significantly different between these groups (*p* = 0.23).

A total of 30 genetic variations in the mitochondrial cytochrome c oxidase subunit 3 (*MT-CO3*) were identified; 22 of them were synonymous variants and eight of them were missense mutations (Table 6).

The percentage of men with total variants in the *MT-CO3* gene groups NS, TS, and CS were: 67.6%, 43.6%, and 45.9% respectively (Table 3). Also, none of these SNPs were significantly different between these groups (*p* = 0.07).

All these variants had been previously reported in the NCBI (https://www.ncbi.nlm.nih.gov/) and in the human mitochondrial DNA database (www.mitomap.org). Except for three novel variants that were found in the *MT-CO3* gene, namely m.9758T > G, m.9657 C > T, and m.9689 A > G (Table 6).

**Table 4:** Genotype frequencies of cytochrome oxidase 1 gene (*MT-CO1*) variants in non-smokers, tobacco-smokers, and cannabis-smokers cases.

SNPs ID	Position	Mutation type	Amino acid change	Genotype	NS (*N* = 37)	TS (*N* = 40)	CS (*N* = 38)
rs1603220215	m.5996 A > G	Synonymous Variant	Thr31	AA AG GG	37 0 0	40 0 0	36 0 2
rs1556423086	m.6152T > A	Synonymous Variant	Val83	TT TA AA	37 0 0	40 0 0	34 0 4
rs1029272	m.6185T > C	Synonymous Variant	Phe94	TT TC CC	36 0 1	37 0 3	38 0 0
rs28439827	m.6329 C > T	Synonymous Variant	Ser142	CC CT TT	36 0 1	40 0 0	38 0 0
rs879112886	m.6026G > A	Synonymous Variant	Leu41	GG GA AA	36 0 1	40 0 0	37 0 1
rs879050330	m.6518 C > T	Synonymous Variant	Gly205	CC CT TT	37 0 0	39 0 1	35 0 3
rs386420010	m.6446G > A	Synonymous Variant	Thr181	GG GA AA	34 0 3	40 0 0	38 0 0
rs370472320	m.6221T > C	Synonymous Variant	Pro106	TT TC CC	37 0 0	39 0 1	38 0 0
rs386829005	m.7337G > A	Synonymous Variant	Ser478	GG GA AA	35 0 2	39 0 1	38 0 0
rs2015062	m.7028 C > T	Synonymous Variant	Ala375	CC CT TT	3 0 34	12 0 28	7 0 31
rs878870695	m.6371 C > T	Synonymous Variant	Ser156	CC CT TT	37 0 0	39 0 1	38 0 0
rs201395766	m.6260G > A	Synonymous Variant	Glu119	GG GA AA	36 0 1	39 0 1	38 0 0
rs2124593224	m.6068 C > T	Synonymous Variant	Asn55	CC CT TT	36 0 1	40 0 0	38 0 0
rs879118820	m.5999T > C	Synonymous Variant	Ala32	TT CT CC	37 0 0	38 0 2	38 0 0
rs879104796	m.6047 A > G	Synonymous Variant	Leu48	AA AG GG	37 0 0	38 0 2	38 0 0
rs1556423059	m.5973G > A	Missense Variant	Ala24Thr	GG GA AA	36 0 1	40 0 0	38 0 0
rs1603220225	m.6018G > A	Missense Variant	Ala39Thr	GG GA AA	37 0 0	39 0 1	38 0 0
rs201262114	m.6261G > A	Missense Variant	Ala120Thr	GG GA AA	37 0 0	34 0 6	38 0 0
rs200165736	m.6253T > C	Missense Variant	Met117Thr	TT TC CC	37 0 0	39 0 1	38 0 0
rs1603220429	m.6340 C > T	Missense Variant	Thr146Ile	CC CT TT	37 0 0	40 0 0	37 0 1
rs879164161	m.6445 C > T	Missense Variant	Thr181Met	CC CT TT	37 0 0	40 0 0	37 0 1
rs1556423267	m.7309T > C	Missense Variant	Ile469Thr	TT TC CC	37 0 0	40 0 0	37 0 1
rs200784106	m.6663 A > G	Missense Variant	Ile254Val	AA AG GG	36 1 0	40 0 0	38 0 0

**Table 5:** Genotype frequencies of cytochrome oxidase 2 gene (*MT-CO2*) variants in non-smokers, tobacco-smokers, and cannabis-smoker’s cases.

SNPs ID	Position	Mutation type	Amino acid change	Genotype	NS (*N* = 37)	TS (*N* = 40)	CS (*N* = 38)
rs1603221136	m.7783T > C	Synonymous Variant	Thr66	TT TC CC	37 0 0	40 0 0	35 0 3
rs1556423330	m.7705T > TC	Synonymous Variant	Tyr40	TT CT CC	36 1 0	40 0 0	37 1 0
rs1603221150	m.7810 C > T	Synonymous Variant	Leu75	CC CT TT	36 1 0	40 0 0	37 1 0
rs386829014	m.7789G > A	Synonymous Variant	Leu68	GG GA AA	36 0 1	40 0 0	37 0 1
rs1556423319	m.7657T > C	Synonymous Variant	His24	TT TC CC	37 0 0	38 0 2	38 0 0
rs368038563	m.7771 A > G	Synonymous Variant	Glu62	AA AG GG	32 0 5	39 0 1	38 0 0
rs879161183	m.7873 C > T	Synonymous Variant	Thr96	CC CT TT	37 0 0	39 0 1	38 0 0
rs1603221066	m.7660T > C	Synonymous Variant	Asp25	TT TC CC	37 0 0	39 0 1	38 0 0
rs1556423316	m.7645T > C	Synonymous Variant	Leu20	TT TC CC	37 0 0	38 0 2	37 0 1
rs879119797	m.7805G > A	Missense variant	Val74Ile	GG GA AA	37 0 0	39 0 1	38 0 0
rs386420037	m.7853G > A	Missense variant	Val90Ile	GG GA AA	36 0 1	40 0 0	38 0 0
rs878897170	m.7830G > A	Missense variant	Arg82His	GG GA AA	37 0 0	40 0 0	37 0 1
rs1556423339	m.7754G > A	Missense variant	Asp57Asn	GG GA AA	37 0 0	40 0 0	37 0 1
rs1603221063	m.7650 C > T	Missense variant	Thr22Ile	CC CT TT	36 1 0	40 0 0	37 1 0
COSV62293493	m.7858 C > T	Synonymous Variant	Asn91	CC TC TT	36 0 1	40 0 0	38 0 0

**Table 6:** Genotype frequencies of cytochrome oxidase 3 gene (*MT-CO3*) variants in non-smokers, tobacco-smokers, and cannabis-smokers cases.

SNPs ID	Position	Mutation type	Amino acid change	Genotype	NS (*N* = 37)	TS (*N* = 40)	CS (*N* = 38)
rs9743	m.9698T > C	Synonymous Variant	Leu164	TT TC CC	34 0 3	39 0 1	36 0 2
rs1603222253	m.9335 C > T	Synonymous Variant	Leu43	CC CT TT	37 0 0	40 0 0	37 0 1
rs374335946	m.9266G > A	Synonymous Variant	Gly20	GG GA AA	37 0 0	39 0 1	35 0 3
rs386829084	m.9548G > A	Synonymous Variant	Gly114	GG GA AA	37 0 0	40 0 0	37 0 1
rs2248727	m.9540T > C	Synonymous Variant	Leu112	TT TC CC	26 0 11	35 0 5	32 0 6
rs372078920	m.9575G > A	Synonymous Variant	Pro123	GG GA AA	36 0 1	40 0 0	38 0 0
rs1556423706	m.9656T > C	Synonymous Variant	Ser150	TT TC CC	34 0 3	39 0 1	37 0 1
rs2124595631	m.9614 A > G	Synonymous Variant	Val136	AA AG GG	36 0 1	40 0 0	38 0 0
rs879237361	m.9530T > C	Synonymous Variant	Pro108	TT TC CC	34 0 3	40 0 0	38 0 0
rs386829079	m.9452G > A	Synonymous Variant	Gly82	GG AG GG	35 0 2	40 0 0	38 0 0
rs2124595636	m.9617 A > G	Synonymous Variant	Leu137	AA AG GG	36 0 1	40 0 0	38 0 0
rs2853824	m.9347 A > G	Synonymous Variant	Leu47	AA AG GG	36 0 1	37 0 3	38 0 0
rs2856985	m.9755G > A	Synonymous Variant	Glu183	GG GA AA	36 0 1	37 0 3	38 0 0
rs375478739	m.9509T > C	Synonymous Variant	Phe101	TT TC CC	37 0 0	39 0 1	38 0 0
rs879028351	m.9758T > C	Synonymous Variant	Ser184	TT TC CC	36 0 1	40 0 0	38 0 0
rs1603222347	m.9497T > C	Synonymous Variant	Phe97	TT TC CC	37 0 0	38 0 2	38 0 0
rs879070193	m.9297 C > T	Synonymous Variant	Leu31	CC CT TT	37 0 0	39 0 1	38 0 0
rs386829074	m.9329G > A	Synonymous Variant	Thr41	GG GA AA	37 0 0	40 0 0	37 0 1
rs28380140	m.9377 A > G	Synonymous Variant	Trp57	AA AG GG	35 0 2	40 0 0	38 0 0
rs879229894	m.9962G > A	Synonymous Variant	Leu252	GG GA AA	37 0 0	40 0 0	35 0 3
rs41482146	m.9667 A > G	Missense Variant	Asn154Ser	AA AG GG	34 0 3	40 0 0	38 0 0
rs2853825	m.9477G > A	Missense Variant	Val91Ile	GG GA AA	35 0 2	40 0 0	38 0 0
rs1556423681	m.9495T > C	Missense Variant	Phe97Leu	TT TC CC	37 0 0	39 0 1	38 0 0
rs1603222339	m.9481T > C	Missense Variant	Phe92Ser	TT TC CC	37 0 0	39 1 0	38 0 0
rs1556423726	m.9801G > A	Missense Variant	Val199Met	GG GA AA	37 0 0	39 0 1	37 0 1
rs1556423714	m.9738G > A	Missense Variant	Ala178Thr	GG GA AA	37 0 0	38 0 2	38 0 0
rs878923250	m.9380G > A	Missense Variant	Trp58Cys	GG GA AA	35 0 2	40 0 0	38 0 0
NOT RECOEDRD/co3	m.9758T > G	Synonymous Variant	Ser184	TT TG GG	36 0 1	40 0 0	38 0 0
NOT RECORDED/co3	m.9657 C > T	Synonymous Variant	Leu151	CC CT TT	37 0 0	39 0 1	38 0 0
NOT RECORDED/co3	m.9689 A > G	Missense Variant	Leu161 Gln	AA AG GG	37 0 0	39 0 1	38 0 0

## Discussion

Since the COVID-19 pandemic, the prevalence of substance use, including tobacco products, marijuana, opioids, and alcohol, has been increasing, especially among men of reproductive age^28,29^. In 2021, 44% of adults surveyed believed smoking marijuana every day is safer than smoking tobacco, compared to about 37% in 2017^30^.

It is widely accepted that unhealthy lifestyles, including consuming alcohol and using tobacco or marijuana products, can negatively affect overall health. However, the trend of increased consumption is concerning because men are often unaware of the negative impact of these lifestyle on their reproductive health. Available evidence suggests that substance use may affect spermatogenesis, secretion of reproductive hormones through the hypothalamic-pituitary-gonadal (HPG) axis, and sexual function^31–36^.

To our best knowledge, this is the first study that aims to compare and determine the effects of tobacco and cannabis smoking in association with variants in the Cytochrome C Oxidase 1, 2 and 3 genes (*MT-CO1*, *MT-CO2*, and *MT-CO3*), on spermatozoa function and male fertility. The findings of the present study showed that normal sperm morphology was significantly lower not only in tobacco smokers but also in cannabis smokers in comparison to non-smokers (*p* < 0.001) (Table 1). Moreover, there was a significant reduction in normal sperm morphology in the cannabis smoker group compared to the tobacco smoker group (*p* = 0.002) (Fig. 1). Sperm progressive motility showed a non-significant reduction in the cannabis-smoking group compared to tobacco-smoking and the non-smoking group (14.27 ± 11.3%) (*p* = 0.223) (Table 1). Moreover, non-progressive motility showed a significant reduction in the cannabis-smoking group and the tobacco-smoking group compared to the non-smoking group (*p* < 0.001). However, the mean percentage of immotile sperm was significantly higher in the cannabis-smoking group compared to tobacco-smoking and the non-smoking group (*p* < 0.001) (Table 1). On the other hand, sperm concentration, and semen volume showed no significant difference between the three groups (*p* = 0.199; *p* = 0.091, respectively) (Table 1).

We investigated the sperm DNA integrity using acridine Orange (AO) assay and Chromomycin staining (CMA3). The results showed a significant increase in the AO + score in the cannabis-smoking group (28.53 ± 15.8%) compared to the non-smoking group (10.1 ± 14.2%) and the tobacco-smoking group (6.4 ± 10.2%) (*p* < 0.001) (Table 2). At the same time, there was no significant difference in AO + score between the tobacco-smoking and non-smoking groups (*p* = 0.19) as shown in Fig. 5. In addition, the CMA3 + test showed a significant increase in the CMA3 + score in the cannabis-smoking group compared to the non-smoking group (*p* < 0.001) and between the tobacco-smoking group compared to the non-smoking group (*p* = 0.003). At the same time, there was a significant increase in the CMA3 + score of cannabis-smoking men in comparison to tobacco-smoking men (*p* = 0.001) (Table 2; Fig. 6).

These results are in accordance with our previous study showing that smoking negatively alters the sperm standard parameters, DNA stability of sperm, and the ratio of protamine mRNA as well as downregulates the expression of H2BFWT, PRM1, and PRM2^36^. Moreover, several studies have reported that the spermatozoa of smokers have higher levels of DNA fragmentation in comparison with non-smokers^36^. Numerous studies have provided insights into the multiple pathways through which tobacco smoking affects sperm cells. For instance, it has been discovered that nicotine impairs spermatogenesis by inducing oxidative stress, DNA damage, and apoptosis within germ cells, leading to reduced sperm count and motility^37,38^.

Also, cigarette smoking has been found to detrimentally impact sperm chromatin condensation and viability. Moreover, these negative effects correlate with both the quantity of cigarettes smoked daily and the overall duration of smoking. In a study conducted by Yu et al. (2014) it was observed that the rate of histone abnormalities was significantly lower in non-smoking men with normal sperm counts, while the highest rates were found in heavy smokers with oligospermia within the Chinese population studied^39^. In addition, excessive ROS production can lead to oxidative stress, which in turn affects sperm nuclear DNA, sperm mitochondrial respiratory activity^40^ and endocrine function, leading to a variety of male reproductive system disorders, and consequently may lead to male infertility^41,42^. In addition, passive smoking effects on various sperm parameters have been studied^43–45^. For example, a decrease in sperm density, motility, and possible negative effects on morphology have been demonstrated^46^. Sperm concentration also decreased by an average of 22% in a dose-dependent manner^47^. Although sperm concentration, motility, and/or morphology are reduced compared to nonsmokers, they generally remain within normal ranges. However, available evidence suggests that smoking may negatively affect sperm binding to the zona pellucida. These findings are based on a study using a penetration test with zona-free hamster eggs^48^.

Cannabis compounds can have a significant impact on sperm motility. ur In our previous study, we have pointed out that the use of high-potency cannabis can lead to a decrease in sperm motility, which may have implications for male fertility^35^. Our findings are consistent with previous reports from fertility centers about the effect of marijuana use on sperm morphology^11,49,50^. In three studies conducted in Jamaica, the Pacific Northwest, marijuana use (past, past 3 months, and current) was associated with an increased risk of abnormal sperm morphology^11,49,50^.

On the other hand, in a study of current, past ever, and never marijuana men users, no significant association with the percentage of normal morphology was found. The risk of morphological abnormalities was also lower compared with men who had never smoked marijuana. Although heavy or recent marijuana use was associated with an increased risk of “abnormal motility” in Jamaican men^49^, no significant associations were found between different categories of marijuana use and total sperm motility percentage^51^.

Nevertheless, a pile of evidence suggested that cannabis smoke impaired male reproductive health, particularly the viability and functionality of sperm. Gundersen et al., documented a significant decrease in sperm concentration among cannabis users compared to non-users^52^.

On the other hand, genetic defects are thought to be the cause of 15–30% of male infertility cases^3^. Several genes associated with fertility and longevity are also associated with mitochondria^26^. Sperm rely on the mitochondrial oxidative phosphorylation machinery (OxPhos) to generate the energy they need for movement^53^.

SNPs or large deletions are types of mutations that affect sperm mitochondrial DNA^54,55^. Therefore, mutated mitochondrial DNA in sperm can cause respiratory problems, affect energy production, and lead to reduced motility, affecting the normal activity of sperm.

Studies have shown that changes in mtDNA may affect sperm velocity and quality^56^. Their study demonstrated that changes in mitochondrial DNA can have a significant impact on sperm quality and its swimming ability^56^.

In the contrary, another study found no significant difference in mtDNA deletions in sperm from asthenozoospermic and normozoospermic men and concluded that these deletions do not have a role in male infertility^57^.

A previous study has reported that mtDNA deletions do not affect sperm motility, as there was no significant difference in the incidence of mtDNA deletions in low-quality and high-quality sperm^58^.

Recently, various researchers showed that the expression of mitochondrial genes can be modulated by lifestyle factors such as smoking and drinking, which also may lead to psychiatric disorders. These habits may affect the activity of the mitochondrial respiratory chain as well as the replication and transcriptional regulation of mitochondrial genes, leading to altered mitochondrial function and, in turn, to psychiatric disorders^59^.

Therefore, this study aimed to investigate the potential genetic alterations within the genes that encode for the mitochondrial cytochrome c oxidase (Complex IV) (*MT-CO1*, *MT-CO2*, and *MT-CO3*) in the sperm cells of individuals who consume tobacco and cannabis. These genes are crucial for cellular respiration and energy production within the cell, and any variations could significantly impact cellular performance. Given the widespread use of tobacco and cannabis, understanding their potential impact on genetic integrity and fertility is important.

A total of 23 SNPs in *MT-CO1* were identified; 15 of them were synonymous variants, while eight were missense mutations (Table 4). The percentage of men with total variants in the *CO1* gene groups NS, TS, and CS were: 97.2%, 82.0%, and 86.4% respectively (Table 3). However, none of these SNPs were significantly different between the studied groups (*p* = 0.10).

A total of 15 genetic alterations in the *MT-CO2* gene were identified; ten of them were synonymous variants and five of them were missense mutations (Table 5). The percentage of men with total variants in the *CO2* gene groups NS, TS, and CS were: 18.9%, 33.3%, and 18.9% respectively (Table 3). None of these SNPs were significantly different between our groups (*p* = 0.23).

A total of 30 genetic variations in MT-CO3 were identified; 22 of them were synonymous variants and eight of them were missense mutations (Table 6). The percentage of men with total variants in the *CO3* gene groups NS, TS, and CS were: 67.6%, 43.6%, and 45.9% respectively (Table 3). Also, none of these SNPs were significantly different between the three groups (*p* = 0.07).

However, other study demonstrated a significantly higher mtDNA mutation in the never-smokers compared to the current-smoker having lung cancer (*p* = 0.006). MtDNA mutation was significantly higher in the never-smoker Asian compared to the current-smoker Caucasian patients’ population (*p* = 0.026). They also observed a significant increase in mtDNA content among the never-smoker lung cancer patients (*p* = 0.037)^60^. Moreover, the US Department of Health and Human Services pointed out that tobacco smokers undergo a substantial build-up of genetic mutations due to the complex chemical composition of tobacco smoke^61^.

Nevertheless, the analysis of sperm exposed to cannabis revealed significant changes in DNA methylation patterns. The hypomethylation of CpG sites observed in individuals who consume cannabis suggests a decrease in methylation of cytosine-phosphate-guanine (CpG) dinucleotides. Tetrahydrocannabinol (THC), the main psychoactive component of cannabis, exerts a variety of effects on the epigenetic properties of sperm^62^. This suggests a possible correlation with the observed differences in mitochondrial DNA (mtDNA). When sperm are exposed to THC, THC interacts with the endocannabinoid system, a complex signalling network that is critical for a variety of physiological activities^13^.

To conclude, the current research demonstrated that cannabis smoking deteriorates sperm quality and DNA integrity more than tobacco smoking. Besides, the lack of a correlation between the identified variant alleles and each of the non-smoker, tobacco smokers and cannabis smoker groups demonstrated that smoking seems unlikely to alter the nucleotide sequence of these genes rather than sperm DNA. However, an analysis of a larger sample size is critical, and would allow a better understanding of the impact of these lifestyles and genetic changes, and could shed light on the risks associated with drug use. The enduring message of this study is the importance of promoting education and awareness about tobacco and marijuana use and their profound effects not only on individual health but also on future generations. Besides, male partners of infertile couples should strictly quit cigarette and cannabis smoking at least three months before undergoing assisted reproductive treatment (ART).

## Methods

### Subjects and study design

One hundred and thirteen semen samples were collected randomly from men of reproductive age attending in vitro fertilization labor (IVF) at Prince Rashid Bin Al Hassan Hospital (PRBH) in Irbid, Jordan. Patients who smoked more than one pack per day for 10 years were considered heavy smokers. Patients who smoked at least 4 joints in a week for more than 3 years were considered cannabis smokers. Thus, we had three groups: Non-smokers (*N* = 37), Tobacco-smokers (*N* = 39), and Cannabis-smokers (*N* = 37).

Moreover, patients older than 40 years old consuming alcohol daily, or having diabetes mellitus, varicocele, chronic disease, recent infection and genetic abnormalities were excluded from the study. Briefly, Human semen samples were obtained by masturbation after three days of sexual abstinence and allowed to liquefy at 37 °C for 30–60 min. Then, the sperm samples were analyzed according to the WHO laboratory manual (WHO, 2010). Semen volume, sperm concentration, morphology, and motility were analyzed across the three groups. This study was performed in accordance with the institutional review board on human experimentation and with the Helsinki Declaration of 1964 and its later amendments and the Jordanian Royal Medical Services-Human Research Ethics Committee approved the project (TF3/1/Ethics Committee/9126). Besides, written informed consent was obtained from all participants.

### Mitochondrial DNA extraction

Before the extraction of sperm DNA, semen samples were loaded on a 45–90% gradient as provided by Nidacon International (Sweden), to purify them from the somatic cells and other debris. The genomic DNA was isolated from the spermatozoa using the QIAamp DNA Mini Kit, followed by mitochondrial DNA amplification via the REPLI-g Mitochondrial DNA Kit supplied by QIAGEN (Hilden, Germany), adhering to the kit’s instruction manual. Then, the purity and quantity of the isolated DNA were checked with a Nanodrop spectrophotometer ND-2000c (Thermo Scientific, USA) and subsequently preserved at -80 °C.

### Polymerase chain reaction (PCR)

To amplify the mitochondrial genes *MT-CO1*, *MT-CO2*, and *MT-CO3*, three sets of polymerase chain reaction (PCR) primers (forward and reverse) were meticulously designed utilizing the Primer 3 software. These primers were designed based on the human mitochondrial sequence procured from the National Centre of Biotechnology Information (NCBI) database (http://www.ncbi.nlm.nih.gov). The oligonucleotide primers were synthesized by Microsynth Seqlab in Germany, as detailed in Table 7.

**Table 7:** Primers list for PCR amplification and Sanger sequencing.

Primer name	Sequence (5′– 3′)	Product length
Mt-Co1 Forward	TCA CCC CCA CTG ATG TTC G	1542 bp
Mt-Co1 Reverse	GGG GGT TCG ATT CCT TCC TTT T	1542 bp
Mt-Co2 Forward	ATA TCT TAA TGG CAC ATG CAG C	684 bp
Mt-Co2 Reverse	GAG GGG GTG CTA TAG GGT AA	684 bp
Mt-Co3 Forward	GCA CGA CAA CAC ATA ATG ACC C	784 bp
Mt-Co3 Reverse	ACT AAA AGA GTA AGA CCC TCA TCA	784 bp
Mt-Co1 Forward Plus*	TTTACAGTAGGAATAGACGTA	*
Mt-Co1 Reverse Plus*	ACCGAAAAATCAGAATAGGTG	*

* Additional internal primers were designed for Sanger sequencing only.

A 25 µL reaction mixture was prepared, comprising 12.5 µL PCR Master Mix (2X) (Thermo Scientific), 0.8 µL of 10 mM forward primer, 0.8 µL of 10 mM reverse primer, 2 µL MT-DNA (20 ng/µL), and 8.9 µL nuclease-free water. The Thermocycler (C1000™ Thermal cycler, Bio-Rad, USA) was used following this program: Initial denaturation at 95 °C for 3 min followed by 35 cycles of denaturation at 95 °C for 30 s. Then, the annealing step for 40 s (Mt-Co1: 59 °C; Mt-Co2 and Mt-Co3: 61 °C), an extension of primers at 72 °C for 1 min, and a final extension for 5 min at 72 °C.

To verify amplification, a subsequent analysis was conducted by running a 5 µL aliquot of each PCR product on a 1% agarose gel stained with GelRed^®^ Nucleic Acid Stain. Visualization was achieved using Molecular Imager Gel Doc XR+ (Bio-Rad, USA).

### Detection of variants in cytochrome C oxidase 1, 2 and 3 (*MT-CO1*, *MT-CO2*, and *MT-CO3*)

The products of PCR were sent for purification and analysis via Sanger sequencing at a local company Microsynth Seqlab, Germany. A bidirectional sequencing (forward and reverse) was conducted for each specimen. Concerning the *MT-CO1* gene, two supplementary internal primers were designed for sequencing (Table 1).

Primary and secondary sequences of every sample were analyzed using the Mutation surveyor (Version 5.2.0), BioEdit sequence alignment editor version 7.2.5, and Unipro UGENE (Version 50.0) software.

### Chromatin condensation evaluation: chromomycin A3 (CMA3) staining

The CMA 3 staining was used to detect the abnormal protamination as described by Manicardi et al., 1995. Slides were fixed using a methanol-glacial acetic acid solution in a (3:1) for 60 min. Then, each slide was treated with 50 µL of CMA3 staining solution and incubated in darkness for 30 min at room temperature (RT). The slides were then rinsed with PBS buffer and mounted with a 1:1 (v/v) PBS/glycerol solution before being stored at 4 °C overnight. At least 300 spermatozoa per slide were examined under a fluorescence microscope: bright green fluorescence (meaning abnormal chromatin packaging) and weak green staining (normal chromatin packaging) of the sperm head. Spermatozoa with a bright green fluorescence in the head are scored as CMA3-positive (CMA3+) and the percentage of CMA3 + sperm is calculated.

### Assessment of sperm DNA fragmentation: Acridine orange (AO) staining

The acridine orange test was to predict sperm DNA damage as described by Tejada et al., 1984. The slides were fixed for a two-hour duration in a freshly constituted Carnoy’s. Then slides were stained with an acidic acridine orange solution. The percentage of spermatozoa with denatured DNA was determined by counting at least 300 spermatozoa under a fluorescent microscope. Spermatozoa with normal, intact double-stranded DNA stained green and those with denatured ones showed red or orange fluorescence. AO-red spermatozoa are scored as AO positive (AO+) and the percentage of AO + sperm is calculated.

### Statistical analysis

All statistical calculations and graphs were generated using GraphPad Prism 6 software. The differences between groups were calculated using the t-test for means, Chi-square, and Fisher exact test for the non-numerical variables. Data are represented as Mean ± SEM (standard error of the mean). The comparisons between the three groups were made using one-way analysis of variance (ANOVA). The *p*-value < 0.05 was considered statistically significant and *p* < 0.01 was highly significant.

## Data Availability

The datasets generated and analysed during the current study are available in the manuscript and in the Genbank repository, PQ868492-PQ868493.
